# Cargo-driven extracellular vesicles as pharmaceutical nanocarriers: A pharmaceutics-oriented comparison of animal exosomes and plant-derived exosome-like nanoparticles

**DOI:** 10.1016/j.ijpx.2026.100530

**Published:** 2026-04-01

**Authors:** Hongfeng Xu, Jin Zhang, Meng Li

**Affiliations:** a College of Biologicaland Chemical Engineering, Jiaxing University, Jiaxing 314001, China; b Jiaxing i-bio Biotechnology Co., Ltd, Jiaxing 314006, China

**Keywords:** Exosomes, Plant-derived exosome-like nanoparticles, Drug delivery systems, MicroRNA and protein cargo, Oral bioavailability, Pharmaceutical translation, Doxorubicin, Corynoxine-B, Curcumin, 5-Fluorouracil, Cholesterol, Sphingomyelin, Phosphatidylserine, Phosphatidylcholine, Monogalactosyldiacylglycerol, Digalactosyldiacylglycerol

## Abstract

Exosomes and plant-derived exosome-like nanoparticles (PELNs) are increasingly investigated as biologically derived nanocarriers that can couple cargo protection with biointerface-enabled transport. From a pharmaceutics standpoint, their therapeutic performance is often cargo-governed (e.g., microRNAs and proteins) and is ultimately constrained by delivery determinants such as stability, biodistribution, cellular uptake, and intracellular trafficking. In this review, we compare animal-derived exosomes (ADEs) and PELNs through a formulation-centric lens, emphasizing how source-dependent molecular composition shapes critical delivery behaviors and translational feasibility. We reorganize representative preclinical evidence into pharmaceutics-relevant delivery scenarios—including systemic/vascular targeting, blood–brain barrier transport, oral gastrointestinal delivery, and tumor microenvironment modulation—to connect cargo identity with exposure-site interactions and pharmacodynamic outcomes. We further discuss engineering strategies for improving payload control, targeting precision, and dosing accuracy, including endogenous enrichment, exogenous loading, and surface functionalization, while highlighting scale-up and safety considerations introduced by modification. Finally, we delineate translational priorities required to advance exosome-based products toward clinical development: standardized dose metrics (particle- and cargo-normalized), quantitative PK/biodistribution–PD relationships, potency assays and critical quality attributes (CQAs), manufacturing consistency under GMP, and regulatory-compliant characterization. Collectively, this review reframes ADEs and PELNs as cargo-driven pharmaceutical delivery systems and provides a practical roadmap for translation, with particular attention to the oral and scalable potential of PELNs.

## Graphical abstract

**Figure:**
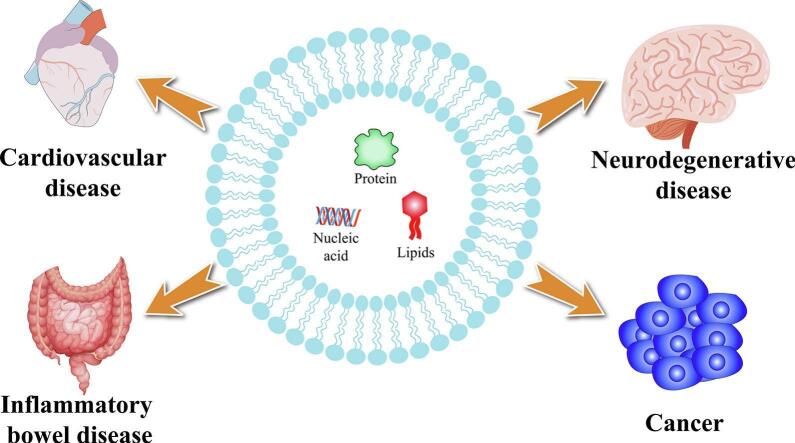

## 1 Introduction

Extracellular vesicles (EVs), including exosomes, are nanoscale, membrane-bound particles released by cells that naturally transport proteins, lipids, and nucleic acids between cells and tissues(Ma et al., 2024). From a pharmaceutics and drug-delivery standpoint, EVs have attracted strong interest as biologically derived nanocarriers that may address persistent limitations of conventional synthetic delivery systems—such as suboptimal biocompatibility, limited barrier penetration, and inefficient delivery to hard-to-reach tissues. EVs exhibit favorable biological interfaces, including low immunogenicity and inherent tissue tropism(Yang et al., 2025), and they can circulate systemically and traverse stringent biological barriers such as the blood–brain barrier(Plavec et al., 2025). These properties position EVs as a promising platform for therapeutic delivery and, in certain contexts, as “nature's nanoparticles” for formulation development.

**Fig. 1:**
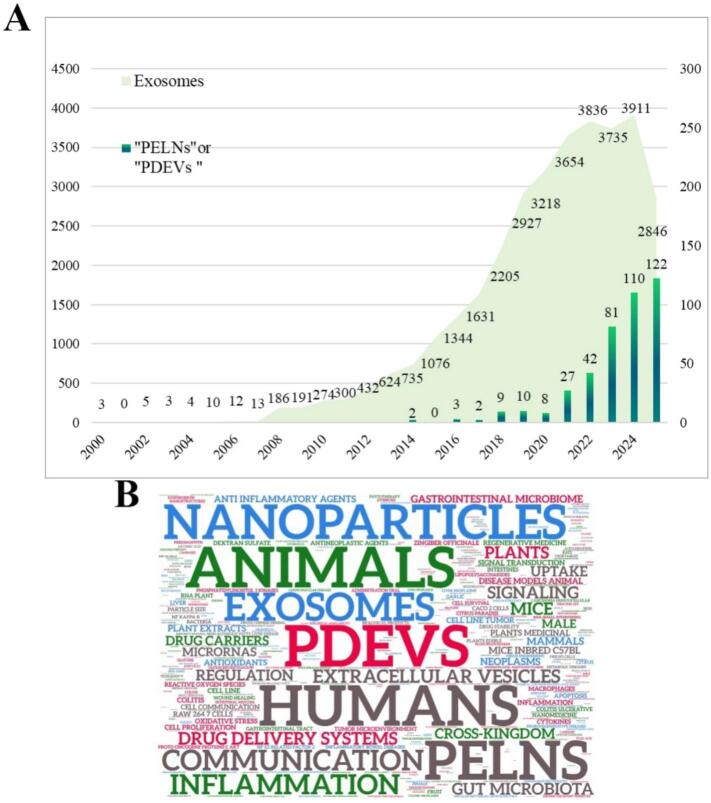
A. Annual number of publications related to exosomes and plant-derived extracellular vesicles indexed in the Web of Science. Exosome-related articles were retrieved using the search term “exosome,” while plant-derived vesicle-related articles were identified using the terms “PELNs” or “PDEVs”. B. Word cloud generated from the titles, abstracts, and keywords of publications related to plant-derived extracellular vesicles and cross-kingdom regulation by plant-derived exosomes，highlighting the most frequently occurring terms.

Consistent with this growing pharmaceutics-driven momentum, bibliometric analyses show a rapid increase in EV-related publications over the past decade (Fig. 1A), with notable expansion in studies focused on both mammalian exosomes and plant-derived extracellular vesicles/exosome-like nanoparticles (PELNs, PDEVs). Keyword co-occurrence analyses further reveal sustained interest at the intersection of EV-based nanomedicine, inflammation, and cross-kingdom communication (Fig. 1B), collectively underscoring the increasing relevance of EVs to translational and pharmaceutical research.

In the broader landscape of nanomedicine, EVs are being explored as drug delivery vehicles and therapeutic agents across diverse disease areas, including cancer, cardiovascular disease, and neurodegenerative disorders(Wang et al., 2025b). These indications remain major global health burdens with substantial unmet clinical needs(Trillos-Almanza et al., 2024), and EV-enabled strategies provide an alternative route to more precise and biologically compatible delivery. For example, engineered exosomes have been used to transport small-molecule drugs or nucleic acids (e.g., siRNAs) to tumor or neuronal cells, leveraging EV-mediated tissue interactions and barrier-crossing potential(Liang et al., 2018). Importantly, EVs may also display intrinsic bioactivity depending on their origin and cargo; mesenchymal stem cell–derived EVs can recapitulate anti-inflammatory and regenerative effects, thereby contributing therapeutic benefit beyond passive carriage(Song et al., 2024).

Despite these advantages, translating EVs into reproducible pharmaceutical products remains challenging. EV preparations are inherently heterogeneous, and robust standardization is complicated by source variability, isolation methods, and cargo diversity. In addition, yields from cell culture can be low, and unmodified EVs often show insufficient tissue-specific targeting, which may limit dose efficiency and therapeutic index(Guo et al., 2025). Accordingly, engineered EVs—modified through cellular, bioengineering, or chemical strategies—are increasingly pursued to improve cargo loading, stability, targeting, and overall pharmacological performance(Cheng et al., 2023). However, these engineering approaches also introduce formulation-centric questions that are central to pharmaceutics, including loading reproducibility, dose control, scale-up compatibility, product characterization, and safety assessment.

In this review, we provide a cargo-centered and pharmaceutics-oriented comparison of ADEs and PELNs, emphasizing how differences in microRNA and protein cargo composition underpin pharmacological activity across disease contexts. We summarize representative preclinical evidence in cardiovascular, neurodegenerative, inflammatory, and oncological models, and discuss how cargo identity and vesicle composition relate to delivery-relevant behaviors such as stability, biodistribution, cellular uptake, and targeting. We further evaluate current strategies to modulate exosomal cargo—endogenous enrichment, exogenous loading, and surface modification—in the context of improving therapeutic efficacy, specificity, and safety. Finally, we also discuss the major challenges and limitations that currently hinder the development of exosome-based nanocarriers, including heterogeneity, dose standardization, loading-related trade-offs, stability, and regulatory uncertainty.

## 2 Biological Characteristics

From a pharmaceutics perspective, the biological characteristics of extracellular vesicles (EVs) directly influence their performance as drug delivery systems, including cargo loading efficiency, stability, biodistribution, cellular uptake, and translational feasibility. Both ADEs and PELNs share a lipid bilayer architecture, yet differ substantially in their biogenesis pathways, molecular composition, and physicochemical properties. These differences underlie distinct formulation behaviors and therapeutic potentials that are highly relevant to pharmaceutical development.

### 2.1 Biogenesis and implications for formulation reproducibility

**Fig. 2:**
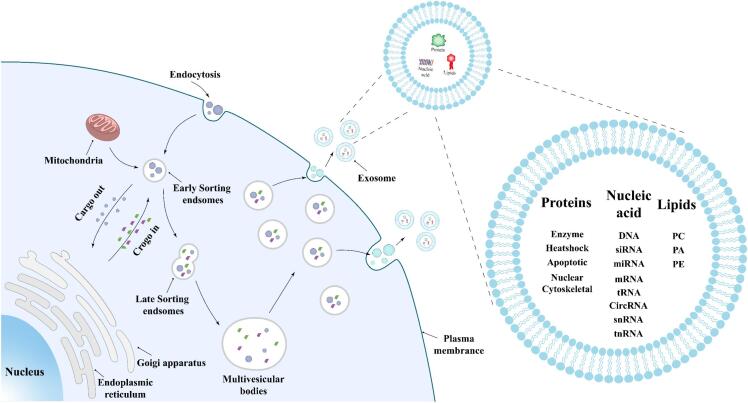
Biogenesis and Composition of Exosomes. Exosomes are formed through a highly regulated endocytic pathway. Cells internalize extracellular substances via endocytosis, generating early endosomes that subsequently mature into multivesicular bodies (MVBs) through the inward budding of their membrane. These MVBs encapsulate specific cargo such as proteins, nucleic acids, and lipids into intraluminal vesicles (ILVs). Upon fusion of MVBs with the plasma membrane, ILVs are released into the extracellular space as exosomes. Exosomes carry diverse bioactive molecules—including RNAs, proteins, and lipids—and participate in intercellular communication, disease biomarker transport, and therapeutic delivery.

ADEs are a major subtype of EVs, typically ranging from 30 to 150 nm in diameter(Igami et al., 2020). Their biogenesis initiates with endocytosis and the formation of early sorting endosomes (ESEs), during which proteins, lipids, mRNAs, and microRNAs are selectively enriched. ESEs subsequently mature into late sorting endosomes (LSEs) under the control of the endosomal sorting complex required for transport (ESCRT) and associated regulatory proteins. Inward budding of the endosomal membrane generates multivesicular bodies (MVBs) containing intraluminal vesicles (ILVs) of approximately 50 nm(McAndrews and Kalluri, 2019). Fusion of MVBs with the plasma membrane ultimately releases ILVs into the extracellular space as exosomes(Williams and Urbé, 2007) (Fig. 2).

From a formulation standpoint, this tightly regulated yet biologically complex biogenesis process contributes to intrinsic heterogeneity in ADE populations. Variations in donor cell type, culture conditions, and intracellular trafficking pathways can result in batch-to-batch variability in size distribution, cargo composition, and surface protein expression(Uceda et al., 2025). Such heterogeneity poses challenges for reproducible manufacturing, quality control, and dose standardization—key considerations in pharmaceutical development.

PELNs represent a heterogeneous population of nanoscale vesicles, generally ranging from 50 to 1000 nm in diameter, and can be isolated from diverse plant materials including fruits, roots, seeds, leaves, and medicinal herbs. Current evidence supports three major biogenetic pathways for PELNs. First, the exocyst-positive organelle (EXPO) pathway, unique to plant cells, mediates cytosolic secretion to the cell wall and has been observed in Arabidopsis and tobacco cells(Wang et al., 2010). Second, MVB-mediated secretion is considered the predominant pathway, analogous to exosome release in mammalian cells(An et al., 2007; Movahed et al., 2019), with vesicle release documented during pathogen–plant interactions(An et al., 2006). Third, vacuolar pathways have been proposed, whereby vesicles originate from vacuoles lacking canonical sorting proteins involved in plant growth and cell wall remodeling(Cui et al., 2019; De Caroli et al., 2021).

Although the molecular regulation of PELN biogenesis remains incompletely understood, their derivation from whole plant tissues rather than cultured cells may confer advantages for large-scale production. In contrast to ADEs, which depend on cell culture–based manufacturing, PELNs can be sourced from renewable agricultural materials, potentially reducing production costs and improving scalability. However, incomplete mechanistic understanding of plant vesicle formation currently limits precise control over cargo composition and uniformity, representing an important knowledge gap for pharmaceutical translation.

### 2.2 Stepwise preparation workflows of ADEs and PELNs for pharmaceutical development

Exosome preparation is a critical upstream determinant of formulation quality because source selection, pretreatment, and recovery workflows jointly influence vesicle yield, purity, cargo composition, and batch-to-batch reproducibility, all of which are central to pharmaceutical development. Although ADEs and PELNs share nanoscale vesicular features, their preparation routes differ substantially due to the distinct nature of cultured mammalian cells versus whole plant tissues.

For ADEs, preparation typically begins with careful donor-cell selection and culture conditioning. Because exosomal cargo and membrane markers depend strongly on the parental cell type and culture state, variables such as passage number, culture medium, oxygen status, and inflammatory stimulation should be controlled as tightly as possible(Ge et al., 2021; Naskou et al., 2024). In most studies, cells are cultured in exosome-depleted serum or serum-free medium for a defined conditioning period, after which the conditioned medium is collected(Pham et al., 2021). The harvested supernatant then undergoes stepwise clarification, usually starting with low-speed centrifugation to remove intact cells, followed by intermediate-speed centrifugation to eliminate cell debris and apoptotic bodies(Wang et al., 2021). The resulting clarified supernatant is subsequently concentrated and subjected to vesicle enrichment. At this stage, differential ultracentrifugation remains one of the most commonly used approaches(Dong et al., 2020), although ultrafiltration- or chromatography-assisted workflows are increasingly adopted to improve scalability and purity(Visan et al., 2022). After enrichment, the vesicle fraction is commonly washed, resuspended in an appropriate buffer such as phosphate-buffered saline, and stored under low-temperature conditions to preserve structural integrity and biological activity(Cheng et al., 2019; Gelibter et al., 2022). From a pharmaceutical perspective, each step in this workflow can alter not only recovery efficiency but also the final composition and functional consistency of the exosome product.

Preparation of PELNs generally follows a different route because the starting material is a complex plant matrix rather than conditioned cell culture medium. The process usually begins with source selection and plant pretreatment, including washing, peeling if necessary, and homogenization or juice extraction from edible or medicinal tissues such as fruits, roots, leaves, or seeds(Garaeva et al., 2021; Zhao et al., 2023). This pretreatment step is particularly important because plant species, tissue type, growth environment, and processing freshness may all influence vesicle yield and compositional heterogeneity(Kilasoniya et al., 2023). After homogenization, large fibers and insoluble particulates are removed by filtration and/or low-speed centrifugation. The clarified plant extract is then subjected to sequential centrifugation to reduce coarse contaminants and enrich nanoscale vesicles. Similar to ADE preparation, downstream recovery and enrichment require further purification steps tailored to the desired balance between yield, purity, and scalability(Huang et al., 2021). The final PELN fraction is resuspended and stored under controlled conditions. Compared with ADEs, PELNs may offer practical advantages for large-scale production because they can be derived from renewable plant materials; however, the complexity of plant matrices also increases the risk of co-isolating non-vesicular components(Xiao et al., 2025), making process standardization especially important(Wang et al., 2025c).

Overall, stepwise preparation workflows are not merely technical details but a major source of variability in exosome-based formulations. Standardizing source selection, pretreatment, clarification, enrichment, washing, and storage is therefore essential not only for improving comparability across studies, but also for providing a more practical methodological reference for future researchers.

### 2.3 Purification-oriented isolation strategies for ADEs and PELNs

In contrast to the upstream preparation workflows described in Section 2.2, which primarily define how vesicle-containing fractions are generated from conditioned medium or plant-derived raw materials, the present section focuses on downstream isolation and, more importantly, purification performance. From a pharmaceutics perspective, the key issue at this stage is not merely vesicle recovery, but the extent to which a given strategy can selectively enrich vesicular populations while minimizing co-isolation of non-vesicular contaminants and preserving structural integrity, compositional consistency, and translational suitability. These considerations are especially important because purification quality directly affects analytical reliability, functional interpretation, cargo engineering, formulation reproducibility, and manufacturing feasibility.

For ADEs, differential centrifugation and ultracentrifugation remain among the most widely used approaches for primary vesicle recovery because of their broad applicability and operational accessibility(Dong et al., 2020). However, although these methods are effective for concentrating vesicle-containing fractions, their selectivity is inherently limited, and co-isolation of protein aggregates, apoptotic bodies, and other extracellular particles may compromise sample purity. In this context, density-gradient centrifugation provides an important refinement by separating vesicles according to buoyant density and can therefore improve purification stringency when higher analytical rigor is required(Zhang et al., 2023). Nevertheless, its prolonged processing time, labor-intensive operation, and limited scalability reduce its practicality for routine large-scale manufacturing. Accordingly, centrifugation-based workflows are often more appropriately regarded as recovery-oriented strategies than as definitive purification solutions.

Compared with centrifugation alone, chromatography- and membrane-based technologies are increasingly favored when purity control, process standardization, and translational compatibility are prioritized. Size-exclusion chromatography (SEC), for example, can effectively separate vesicles from soluble proteins and low-molecular-weight contaminants under relatively mild conditions, thereby helping preserve vesicle integrity and biological activity(An et al., 2018). Likewise, ultrafiltration and tangential flow filtration (TFF) have attracted growing interest because they enable simultaneous concentration and partial purification within a more scalable and process-compatible framework(Visan et al., 2022). These advantages are particularly relevant to pharmaceutical development, where throughput, reproducibility, and integration into standardized manufacturing workflows are essential. However, membrane-based methods are not without limitations, as membrane fouling, vesicle adsorption, and incomplete exclusion of similarly sized impurities may adversely affect both recovery and purity. Polymer-based precipitation, although simple and accessible, generally exhibits inferior selectivity and often leads to co-enrichment of non-vesicular materials, thereby limiting its suitability for applications requiring rigorous quality control(Morozumi et al., 2021). By contrast, immunoaffinity capture and microfluidic platforms provide enhanced specificity and are particularly attractive for analytical and diagnostic applications, although their current limitations in throughput, cost, and scalability remain major barriers to broader use in therapeutic manufacturing(Cipa et al., 2024).

For PELNs, downstream purification is intrinsically more challenging because the starting matrix is substantially more heterogeneous than that of ADEs. Plant-derived materials contain abundant non-vesicular constituents, including cell wall debris, fibrous structures, pigments, soluble metabolites, and other colloidal components, all of which may be co-enriched during vesicle isolation(Huang et al., 2021). Consequently, the principal challenge in PELN purification is not simply vesicle recovery, but effective decontamination of a highly complex biological matrix. This distinction is particularly important because purification strategies that are acceptable for ADEs may prove insufficiently selective when directly transferred to plant-derived systems. Although density-based separation and filtration-assisted approaches can improve product cleanliness, their performance is strongly influenced by plant source, tissue composition, and preprocessing conditions, and the complexity of heterogeneous plant matrices may further increase process difficulty(Xiao et al., 2025). Therefore, in PELN research, purification stringency should be regarded not merely as a technical refinement but as a prerequisite for batch consistency, physicochemical interpretability, and credible assignment of downstream biological effects.

Taken together, no single isolation and purification strategy can be considered universally optimal for either ADEs or PELNs. Rather, method selection should be guided by the intended application and by the balance required among recovery efficiency, purity, vesicle integrity, throughput, and scalability(Visan et al., 2022). For exploratory studies, conventional centrifugation-based workflows may remain acceptable because of their practicality and widespread accessibility, whereas SEC-, ultrafiltration-, and TFF-assisted workflows appear more promising for pharmaceutical translation and scalable production(Grenhas et al., 2024). In the case of PELNs in particular, minimizing co-isolation of plant-derived non-vesicular components should be regarded as a primary purification objective rather than a secondary technical consideration, because these impurities may compromise both batch consistency and biological interpretability(Wang et al., 2025c; Xiao et al., 2025). Therefore, the establishment of standardized and source-appropriate purification workflows is essential for reliable characterization, reproducible cargo engineering, robust quality control, and eventual clinical translation.

### 2.4 Structural composition and formulation-relevant properties

Following preparation, isolation, and purification, both ADEs and PELNs exhibit distinct compositional features that are closely related to their biological origin and processing workflow (Table 1). ADEs are enriched in characteristic membrane proteins such as tetraspanins (CD9, CD63, CD81), heat shock proteins, ESCRT-associated proteins, and signaling molecules, as well as nucleic acids including mRNAs and microRNAs (Kugeratski et al., 2021). Their lipid membranes are rich in cholesterol, sphingomyelin, and phosphatidylserine, which contribute to membrane rigidity and prolonged circulation stability.

**Table 1:** Composition Differences between PELNs and ADEs.

Category	ADEs	PELNs	Ref.
Lipid	Cholesterol, Sphingolipids, Phosphatidylserine, Phosphatidylethanolamine (PE), Phosphatidylcholine (PC), Phosphatidylinositol	Phosphatidylethanolamine, Phosphatidyl acid (PA), Phosphatidylcholine, Digalactosyl monoacylglycerol (DGMG), Digalactosyl diacylglycerol (DGDG), Monogalactosyl diacylglycerol (MGDG)	(Ju et al., 2013; Skotland et al., 2019; Zhang et al., 2016)
Protein	Membrane transporters, Fusion proteins, Chaperones, Cytoskeletal proteins, MVBs, Synthesis proteins, Tetraspanins, adhesion molecules	Aquaporins, ATPases, ABC transporters, Tetraspanins, reticulin heavy chains, heat shock proteins	(Pocsfalvi et al., 2018; Stanly et al., 2019; Wang et al., 2014)
Nucleic acid	DNA, mRNA, miRNA, SnoRNA, lncRNA	DNA, mRNA, miRNA, non-coding RNA	(Aquilano et al., 2019; O'Brien et al., 2020; Teng et al., 2018)
Small molecule	Not available	Vitamin C, naringenin, 6-gingerol, and 6-shogaol	(Perut et al., 2021; Zhang et al., 2016)

PELNs, in contrast, contain plant-specific lipids such as phosphatidylcholine, digalactosyldiacylglycerol, and monogalactosyldiacylglycerol, along with proteins, polysaccharides, secondary metabolites, and plant-derived microRNAs (Ma et al., 2025). These compositional differences influence physicochemical properties such as membrane fluidity, enzymatic stability, and interactions with biological membranes. Notably, the lipid composition of PELNs may enhance resistance to gastrointestinal degradation, supporting their exploration as orally administered delivery systems.

From a pharmaceutics standpoint, cargo composition is not merely a biological descriptor but a determinant of formulation performance. Encapsulation within a lipid bilayer protects nucleic acids and proteins from enzymatic degradation, enhances in vivo stability, and influences release kinetics(Wu et al., 2025). Moreover, the presence of bioactive lipids and metabolites in PELNs raises the possibility of synergistic pharmacological effects, in which the vesicle functions both as a carrier and as an active component of the formulation. These compositional features not only influence biological function, but also define the key parameters that require systematic characterization during formulation development.

### 2.5 Characterization strategies and formulation-relevant quality attributes

Characterization is a core component of exosome formulation development because it helps define and verify the quality attributes that govern performance, reproducibility, and translational feasibility. For both ADEs and PELNs, characterization should extend beyond descriptive identification alone to include systematic evaluation of physicochemical properties, molecular composition, cargo incorporation, stability, and downstream functional performance. These parameters collectively help define the critical quality attributes of exosome-based formulations(Dong et al., 2020; Gelibter et al., 2022).

At the physicochemical level, particle size, size distribution, concentration, surface charge, and morphology are among the most fundamental parameters. Techniques such as nanoparticle tracking analysis (NTA), dynamic light scattering (DLS), zeta potential analysis, and transmission electron microscopy (TEM) are commonly used to assess vesicle size, colloidal behavior, and structural integrity. These parameters are formulation-relevant because they influence dispersion stability, cellular uptake, biodistribution, and batch consistency(Cheng et al., 2019).

Compositional characterization is equally important. For ADEs, protein markers, nucleic acid profiles, and lipid composition are commonly used to verify vesicle identity and membrane properties, often through approaches such as Western blotting, proteomics, qPCR, sequencing, and lipidomic analysis(Keysberg et al., 2021; Kugeratski et al., 2021). For PELNs, characterization should also consider plant-specific lipids, metabolites, and the possible co-isolation of non-vesicular plant-derived components. Accordingly, proteomic, lipidomic, and nucleic acid analyses are important for linking vesicle composition to biological activity and formulation quality, particularly in distinguishing vesicle-associated components from co-isolated plant-derived impurities(Ma et al., 2025).

From a drug-delivery perspective, loading-related characterization is also essential. Parameters such as loading efficiency, cargo retention, release behavior, and functional activity should be evaluated together with stability under relevant storage and processing conditions(O'Brien et al., 2022). Depending on cargo type, fluorescence-based assays, qPCR, or chromatographic methods may also be used for quantitative evaluation. As development advances toward translational application, batch consistency, residual impurities, and process-related quality attributes become increasingly important(Gelibter et al., 2022). Overall, characterization should be regarded as an integral component of formulation development, as it directly supports formulation optimization, quality control, and translational evaluation.

### 2.6 Cellular transport and uptake mechanisms

The therapeutic performance of exosome-based formulations depends critically on their interactions with recipient cells. ADEs exhibit selective uptake by specific cell types, mediated by surface proteins, proteoglycans, and lipids that engage complementary receptors on target cells(Christianson et al., 2013; Desrochers et al., 2016; Rana et al., 2012). Following surface binding, ADEs can enter cells via direct membrane fusion or multiple endocytic pathways, including clathrin-dependent endocytosis (CDE) and clathrin-independent endocytosis (CIE)(Svensson et al., 2013). CIE pathways encompass macropinocytosis, phagocytosis, and lipid raft–mediated uptake, all of which influence intracellular trafficking and cargo release(Romenskaja et al., 2024).

PELNs display similarly diverse uptake mechanisms, including membrane fusion, receptor-mediated endocytosis, and lipid raft–dependent internalization. For example, garlic-derived nanovesicles interact with CD98 on hepatocytes to trigger uptake(Song et al., 2020), while plant-derived sphingolipids have been implicated in lipid raft–mediated internalization(Izquierdo-Useros et al., 2009). Additional mechanisms such as clathrin-mediated endocytosis, caveolae-dependent uptake, macropinocytosis, and microtubule-dependent transport have also been reported(Kim et al., 2020; Regente et al., 2017; Sasaki et al., 2021; Wang et al., 2014; Zhuang et al., 2015).

These uptake pathways have direct implications for drug delivery efficiency, tissue targeting, and intracellular bioavailability. Importantly, the surface chemistry and lipid composition of PELNs may favor accumulation in inflamed or diseased tissues, particularly within the gastrointestinal tract, supporting their development as orally administered nanocarriers. Understanding and controlling these transport mechanisms is therefore central to optimizing exosome-based formulations.

### 2.7 Pharmaceutical advantages of PELNs

Although exosomes from different biological origins share common structural features, their suitability for pharmaceutical applications differs substantially. Among available EV platforms, PELNs exhibit several formulation-relevant advantages that position them as attractive candidates for next-generation drug delivery systems.

#### 2.7.1 Sustainable and scalable production

PELNs can be isolated from a wide range of edible plants (e.g., lemon, grape, mushroom) and medicinal herbs (e.g., ginger, ginseng), enabling cost-effective and scalable production(Adamo et al., 2021; You et al., 2021). In contrast to ADEs, which often rely on limited biological sources such as milk, blood, or cultured cells, plant materials can be cultivated at agricultural scale with relatively low manufacturing costs(Bian et al., 2022; Shokravi et al., 2022). This scalability is particularly attractive for chronic or oral therapies requiring repeated dosing.

#### 2.7.2 Safety and biocompatibility

PELNs generally exhibit high biocompatibility and low immunogenicity, reducing the risk of adverse immune responses(Xiao et al., 2022). In addition, they are free from zoonotic pathogens, mitigating biosafety concerns associated with animal-derived materials(Cao et al., 2019). These properties support their potential use in oral formulations and may simplify regulatory considerations compared with mammalian EVs.

#### 2.7.3 Intrinsic pharmacological activity

PELNs derived from phytochemical-rich plants may encapsulate bioactive metabolites with underexplored therapeutic potential(Salmerón-Manzano et al., 2020). For example, PELNs from ginseng, ginger, grape, and honeysuckle may carry ginsenosides, curcuminoids, flavonoids, and other secondary metabolites(Ju et al., 2013; Teng et al., 2018; Xu et al., 2021; Zhou et al., 2020). Encapsulation within vesicles may protect these compounds from degradation and enhance their bioavailability, allowing PELNs to function as multifunctional formulations that combine delivery and pharmacological activity. This dual role distinguishes PELNs from conventional inert carriers and highlights their promise as biologically active drug delivery platforms.

## 3 Therapeutic applications

**Fig. 3:**
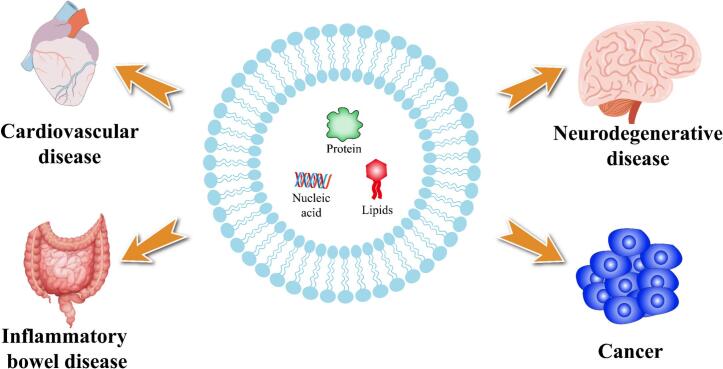
Exosome-based delivery scenarios and representative therapeutic endpoints. Exosomes deliver diverse bioactive cargo—including proteins, nucleic acids (e.g., DNA, mRNA, and miRNAs), lipids, and exogenously loaded therapeutics—enabling multiple pharmaceutics-relevant delivery scenarios. Representative scenarios include systemic delivery with vascular/lesion targeting, central nervous system delivery across the blood–brain barrier, oral delivery with gastrointestinal site targeting, and tumor delivery accompanied by microenvironmental and immune modulation. Depending on cargo identity and source, exosomes may function as biologically derived nanocarriers and/or intrinsically active formulations, supporting therapeutic intervention and, in some contexts, biomarker readouts.

Exosomes and exosome-like nanoparticles can be viewed as biologically derived delivery systems whose therapeutic performance is governed by both molecular cargoes and membrane-associated determinants, including miRNAs, proteins, lipids, and other bioactive constituents, together with delivery-relevant behaviors such as stability, biodistribution, cellular uptake, and barrier traversal. Across indications, reported applications can be reorganized into four pharmaceutics-oriented delivery scenarios: (i) systemic delivery with vascular/lesion targeting, (ii) central nervous system (CNS) delivery across the blood–brain barrier (BBB), (iii) oral delivery with gastrointestinal (GI) site targeting, and (iv) tumor delivery with microenvironmental and immune modulation. This section summarizes representative preclinical evidence within each delivery scenario (Fig. 3).

### 3.1 Systemic delivery and vascular targeting

#### 3.1.1 Anti-atherosclerotic and antithrombotic delivery endpoints

In cardiovascular settings, circulating and tissue-derived exosomes have been implicated in both protective and pathogenic processes, highlighting the importance of delivery context and cargo composition. Platelet-derived exosomes were reported to inhibit thrombosis in atherosclerosis by reducing CD36-dependent lipid accumulation in macrophages and suppressing platelet aggregation (Srikanthan et al., 2014). In contrast, exosomes from vascular smooth muscle cells (VSMCs) may promote vascular calcification and thrombosis by transferring γ-carboxyglutamic acid–containing proteins involved in coagulation-related processes (Kapustin et al., 2017). From a drug-delivery standpoint, these examples collectively underscore that exosomal surface/cargo features can modulate vascular cell uptake and downstream coagulation/inflammation pathways, which should be considered when designing systemic exosome-based formulations for vascular indications.

#### 3.1.2 Myocardial repair/regeneration as a delivery objective

In regenerative contexts, exosomes derived from embryonic stem cells and induced pluripotent stem cells have been reported to enhance cardiomyocyte proliferation, angiogenesis, and survival(Fijnvandraat et al., 2003). Exosomes from cardiac stem cells and mesenchymal stem cells can support repair by modulating immunity, promoting neovascularization, and reducing fibrosis(Yuan et al., 2018). Mechanistically, miR-21 in cardiac progenitor cell exosomes protects cardiomyocytes by targeting PDCD4(Xiao et al., 2016), and MSC-derived exosomal miR-22 promotes repair by targeting MECP2(Feng et al., 2014). Human MSC-derived exosomal miR-21-5p was further shown to improve calcium handling and mitochondrial function by regulating sarco/endoplasmic reticulum Ca^2+^-ATPase and L-type calcium channels, thereby improving cardiac contractility(Mayourian et al., 2018). These studies position exosomes as carriers of functional RNA/protein cargo capable of driving repair-relevant pharmacodynamic endpoints following systemic or local exposure, while also motivating more quantitative characterization of dose–response relationships and exposure–effect coupling for translational development.

#### 3.1.3 PELNs for vascular inflammation: Stability and lesion-preferential accumulation

PELNs have also been explored in cardiovascular applications, where formulation-relevant properties such as cargo stability and site-preferential accumulation may be advantageous. For example, green tea–derived nanovesicles loaded with a piRNA antagonist improved cargo stability and enabled preferential accumulation at aortic dissection sites(Liu et al., 2024). In addition, PELNs have been suggested to promote macrophage polarization toward an M2 phenotype and inhibit VSMC osteogenic transdifferentiation, thereby reducing vascular calcification(Feng et al., 2023). Together, these findings support the feasibility of PELNs as systemic nanocarriers for vascular lesions, while highlighting the need to define delivery determinants (e.g., stability in circulation and lesion-retentive interactions) that drive preferential tissue exposure.

### 3.2 Central nervous system delivery across the blood–brain barrier

#### 3.2.1 Blood–Brain Barrier (BBB) traversal and the dual role of exosomes in neurodegeneration

In neurodegenerative disorders, exosomes may exert dual roles: propagating pathogenic proteins or serving as therapeutic carriers(Deng et al., 2017a; Saman et al., 2012). In prion diseases and Alzheimer's disease (AD), exosomes can carry misfolded prion protein (PrP^Sc^), amyloid-β (Aβ), and hyperphosphorylated tau, thereby contributing to disease progression(Asai et al., 2015; Guo et al., 2016; Iyaswamy et al., 2023; Zhao et al., 2022). From a delivery perspective, these observations illustrate that exosome-mediated intercellular trafficking is a biologically enabled transport route within the CNS, but that cargo selection is critical to ensuring therapeutic (rather than pathogenic) outcomes.

#### 3.2.2 Therapeutic CNS delivery examples organized by delivery objective

Exosomes have been engineered as CNS carriers to enhance intracellular delivery and improve disease-relevant outcomes. Engineered neuronal exosomes loaded with Corynoxine-B were reported to enhance delivery to diseased neurons, promote autophagy, and ameliorate cognitive deficits in Alzheimer's models(Iyaswamy et al., 2023). In Parkinson's disease (PD), while exosomes may contribute to α-synuclein transmission, MSC-derived exosomes may provide neuroprotection by crossing the BBB, modulating inflammation, and delivering therapeutic miRNAs(Guo et al., 2020; Heris et al., 2022; Jiang et al., 2020). In parallel, carrot-derived nanovesicles were reported to activate antioxidant pathways—including Nrf2, HO-1, and NQO1—thereby reducing reactive oxygen species and neuronal apoptosis(Kim and Rhee, 2021).

These studies collectively emphasize BBB traversal and neuronal/glial uptake as key delivery bottlenecks, and they motivate systematic evaluation of CNS biodistribution, intracellular trafficking, and cargo release as part of pharmaceutics-driven development.

#### 3.2.3 ALS: Toxic cargo transport, biomarker potential, and repair-focused delivery

In amyotrophic lateral sclerosis (ALS), astrocyte-derived exosomes can transport toxic proteins such as Cu/Zn-superoxide dismutase (SOD1), thereby contributing to motor neuron injury(Basso et al., 2013). Additionally, miRNA profiles in serum- and plasma-derived exosomes may reflect disease progression and serve as candidate diagnostic biomarkers(Banack et al., 2020; Saucier et al., 2019). Therapeutically, exosomes from adipose-derived stem cells have shown potential by homing to damaged regions, supporting motor neuron and neuromuscular junction repair, modulating mitochondrial function, and reducing glial overactivation(Bonafede et al., 2020).

### 3.3 Oral delivery and gastrointestinal targeting

#### 3.3.1 Dietary/food-derived EVs as oral formulations: Stability and microbiome-associated endpoints

Oral administration offers a distinctive opportunity for EV-based delivery because local intestinal exposure may be sufficient to elicit therapeutic effects. In IBD-related studies, milk-derived exosomes were reported to influence host physiology and microbial composition. Diets enriched with exosomes and RNA altered the cecal microbiota of C57BL/6 mice, increasing beneficial taxa such as Tenericutes and Lachnospiraceae (Zhou et al., 2019). Mechanistically, milk exosomes enhanced barrier function by upregulating protective genes (e.g., Muc2, RegIIIγ, Myd88, and GATA4) and increasing IgA and secretory IgA levels. They also promoted beneficial microbes (e.g., Akkermansia, Muribaculum, and Turicibacter) while suppressing potentially harmful taxa such as Desulfovibrio, thereby restoring microbial homeostasis(Tong et al., 2020). From a pharmaceutics perspective, these outcomes highlight oral EV stability and mucosal interactions as formulation-relevant determinants of local bioactivity.

#### 3.3.2 PELNs for colitis: Inflamed-site targeting and mucosal repair

PELNs have been reported to preferentially target inflamed intestinal regions and alleviate colitis through multiple mechanisms, including suppressing pro-inflammatory cytokines, enhancing anti-inflammatory responses, maintaining barrier integrity, and promoting intestinal stem cell proliferation. Grape-derived nanoparticles stimulate intestinal stem cell renewal and mucosal regeneration and alleviate DSS-induced colitis(Ju et al., 2013). Oral administration was reported to enhance epithelial survival and proliferation, downregulate pro-inflammatory cytokines, and increase anti-inflammatory cytokines, thereby mitigating colonic inflammation(Zhang et al., 2016). Ginger-derived PELNs suppress inflammatory mediators (e.g., TNF-α, IL-6, and IL-1β) while upregulating HO-1 via inhibition of NF-κB signaling(Liu et al., 2022). Broccoli-derived PELNs have also been reported to inhibit dendritic cell activation and promote tolerogenic dendritic cells, thereby reducing TNF-α and IL-17 in experimental colitis models(Deng et al., 2017b).

### 3.4 Oncology: Tumor delivery, immune modulation, and combination-oriented outcomes

#### 3.4.1 Tumor delivery barriers and metastasis-associated transport effects

Exosomes regulate key steps of cancer progression and metastasis, including angiogenesis, tumor cell migration, epithelial–mesenchymal transition (EMT), and pre-metastatic niche formation(Pucci et al., 2023; Ridder et al., 2015; Yang et al., 2024; Zeng et al., 2018). Tumor-derived exosomes facilitate metastasis by delivering pro-metastatic cargo that remodel the tumor microenvironment(Wortzel et al., 2019). For example, pancreatic cancer exosomal circ-IARS increases endothelial permeability by activating RhoA signaling and downregulating ZO-1, thereby promoting intravasation and vascular dissemination(Li et al., 2018). Ovarian cancer exosomal miR-205 was reported to promote angiogenesis and accelerate disease progression(He et al., 2019). Similar exosome-mediated effects have been implicated in nasopharyngeal carcinoma, hepatocellular carcinoma, and renal cell carcinoma(Boonkaew et al., 2023; Chen et al., 2022; He et al., 2023; Li et al., 2022). In a delivery-centric framing, these studies illustrate how exosomal cargo can modulate vascular permeability and stromal conditions that ultimately influence both tumor dissemination and the exposure landscape for therapeutic delivery.

#### 3.4.2 Immune modulation as a delivery-relevant outcome affecting therapeutic index

Beyond metastasis, exosomes also contribute to tumor immune evasion. Programmed death-ligand 1 (PD-L1) displayed on tumor-derived exosomes can suppress systemic antitumor immune responses(Poggio et al., 2019). Depletion of exosomal PD-L1 inhibits tumor growth, including in models resistant to PD-L1 antibody therapy. For example, melanoma exosomal PD-L1 impairs CD8^+^ T cell function and promotes tumor progression(Chen et al., 2018). In prostate cancer, exosomes carrying prostaglandin E2 induce CD73 expression on dendritic cells, increasing extracellular adenosine and suppressing CD8^+^ T cell–mediated immunity(Salimu et al., 2017). In addition, melanoma-derived exosomes containing miRNAs (e.g., hsa-miR-3187-3p and hsa-miR-498) impair T cell receptor signaling and TNF-α production(Vignard et al., 2020), whereas exosomes from tumor-associated macrophages in epithelial ovarian cancer disrupt the Treg/Th17 balance via miR-29a-3p and miR-21-5p to promote immune escape(Zhou et al., 2018). Collectively, these findings indicate that exosomal surfaces/cargo can systemically reshape immune tone, thereby affecting the therapeutic index of anticancer delivery strategies and supporting the rationale for immune-aware design of exosome-based formulations.

#### 3.4.3 PELNs as carriers and intrinsically active formulations

Given their low immunogenicity, biodegradability, and minimal toxicity, PELNs have shown potential in cancer prevention and therapy. *Moringa oleifera* seed–derived vesicles carry miRNAs that regulate tumor proliferation and apoptosis while eliciting minimal immune responses(Potestà et al., 2020). PELNs may also help overcome drug resistance; bitter melon–derived vesicles sensitize oral squamous cell carcinoma cells to 5-fluorouracil by downregulating NLRP3 and increasing ROS(Yang et al., 2021). Notably, some PELNs exhibit intrinsic anticancer activity, potentially due to enrichment in plant-derived bioactives and multimodal mechanisms. Lemon-derived vesicles induce S-phase arrest and apoptosis in gastric cancer cells via ROS-associated pathways(Yang et al., 2020). *Panax ginseng*–derived vesicles suppress melanoma growth by reprogramming tumor-associated macrophages through TLR4–MyD88 signaling(Cao et al., 2019). Moreover, vesicles derived from *D. morbifera, P. densiflora, T. occidentalis,* and *C. obtusa* show selective cytotoxicity across cancer cell lines, with D. morbifera and *P. densiflora* vesicles exhibiting strong anticancer activity while sparing normal cells(Kim et al., 2020). Representative engineered exosome-based drug delivery systems discussed in this section are summarized in Table 2 to facilitate comparison of disease setting, source, cargo, engineering strategy, therapeutic outcome, and reference.

**Table 2:** Representative exosome-based drug delivery systems in major therapeutic settings.

Disease	Source	cargo	Engineering strategy	Outcome	Ref.
Aortic dissection / vascular inflammation	Green tea leaves	piRNA antagonist	Cargo loading into plant-derived nanovesicles	Improved lesion-targeted delivery	(Liu et al., 2024)
Alzheimer's disease	Engineered neuronal cells	Corynoxine-B	Cargo loading into engineered neuronal exosomes	Enhanced neuronal delivery and cognition	(Iyaswamy et al., 2023)
Solid tumors	Tumor-cell-derived	Doxorubicin	Genetic surface display of iRGD-LAMP-2B	Enhanced tumor targeting and efficacy	(Tian et al., 2014)
CNS-targeted RNA delivery	Engineered exosomes	siRNA	Surface display of rabies virus glycoprotein (RVG)	Enhanced brain delivery and BACE1 knockdown	(Alvarez-Erviti et al., 2011)
Tumor-directed nucleic acid delivery	HEK293T cells	miRNA / CRISPR-dCas9-related RNA cargo	Donor-cell engineering with CD9-HuR functionalization	Improved RNA loading and delivery	(Li et al., 2019)
Cancer immunotherapy	Engineered exosomes	CpG oligonucleotide cargo	Genetic engineering of CpG-SAV-Exo	Enhanced antitumor immune response	(Morishita et al., 2016)

## 4 Current advances in exosome engineering

**Fig. 4:**
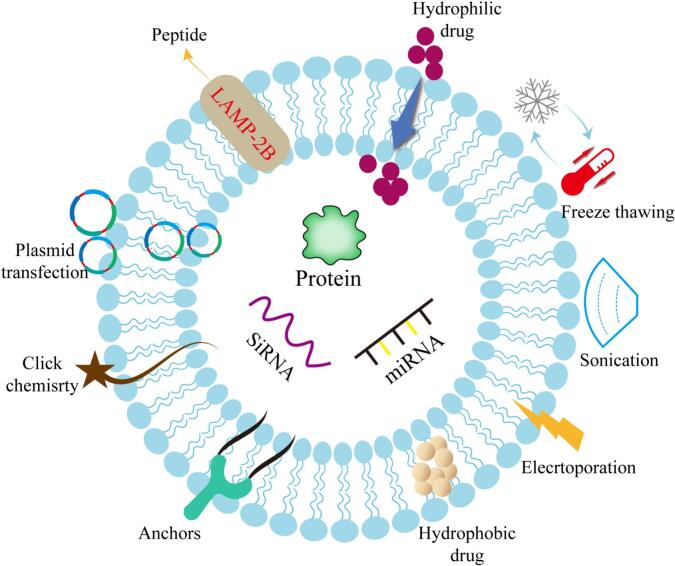
Engineering strategies for exosome-based drug delivery. Exosome engineering mainly involves cargo loading and surface modification. Cargo loading can be achieved through physical, chemical, or biological strategies, including co-incubation, electroporation, sonication, freeze–thaw cycling, extrusion, CaCl₂-mediated loading, chemical-facilitated loading, and endogenous loading through donor-cell transfection or engineering. Surface modification can be introduced by ligand conjugation, membrane insertion, or genetic engineering to enhance targeting specificity and cellular uptake. These strategies collectively improve drug-loading capacity, delivery precision, and the therapeutic potential of exosome-based nanocarriers.

Recent advances in exosome engineering have substantially expanded the utility of exosomes as drug delivery platforms (Kim et al., 2024). Owing to their intrinsic biocompatibility and membrane stability, exosomes provide a favorable basis for further engineering aimed at improving cargo loading, targeting precision, and therapeutic performance (Bahadorani et al., 2024). Current engineering strategies mainly involve cargo-loading approaches and surface-modification approaches. Depending on cargo properties and therapeutic requirements, cargo loading may be achieved through physical, chemical, or biological methods, whereas surface engineering is commonly employed to improve tissue targeting and cellular uptake(Zeng et al., 2023a; Zhao et al., 2024). Together, these strategies improve the flexibility and translational potential of exosome-based delivery systems (Fig. 4).

### 4.1 Cargo loading strategies

Exosomes can serve as versatile delivery vehicles for diverse therapeutic cargo, including proteins, nucleic acids, and small molecules. The lipid bilayer protects cargo from enzymatic degradation, thereby improving in vivo stability and bioavailability. To maximize their utility as delivery vehicles, efficient cargo-loading strategies are required. Current approaches can be broadly divided into exogenous loading, in which cargo is introduced after exosome isolation, and endogenous loading, in which cargo is enriched during exosome biogenesis within donor cells. From a practical formulation perspective, exogenous loading methods can be further classified into physical and chemical approaches, whereas endogenous loading corresponds primarily to biological loading strategies.

#### 4.1.1 Physical loading methods

Physical loading methods introduce cargo into isolated exosomes by exploiting passive membrane partitioning or temporarily disturbing membrane integrity. These approaches are widely used because they are relatively straightforward and can be adapted to different classes of therapeutic molecules.

A simple physical strategy is co-incubation, which is particularly suitable for hydrophobic or membrane-permeable molecules(Brossa et al., 2021). In a typical procedure, purified exosomes are mixed with the therapeutic cargo at a defined ratio and incubated for a fixed period under controlled temperature conditions, allowing the cargo to partition into or associate with the lipid bilayer. Unencapsulated molecules are then removed by ultracentrifugation, ultrafiltration, or size-exclusion purification. This method is operationally simple and preserves vesicle structure well, but loading efficiency is often limited, especially for hydrophilic molecules and large nucleic acids(Mukhopadhya et al., 2023; Nemidkanam et al., 2024).

Electroporation is one of the most commonly used physical approaches for loading nucleic acids such as siRNAs and miRNAs(Pomatto et al., 2019). In general, isolated exosomes are suspended together with the desired nucleic acid cargo in an appropriate conductive buffer, followed by application of short electrical pulses to transiently permeabilize the vesicle membrane. After pulse treatment, the mixture is incubated briefly to allow membrane resealing, and free nucleic acids or aggregates are subsequently removed. Electroporation can improve nucleic acid loading efficiency, but it may also induce vesicle aggregation, membrane destabilization, or nucleic acid precipitation if conditions are not carefully optimized(Torabi et al., 2024).

Sonication enhances loading by transiently deforming the exosomal membrane through mechanical shear. Typically, purified exosomes are mixed with the therapeutic cargo and subjected to several short sonication cycles interspersed with cooling periods to avoid overheating(Mukhopadhya et al., 2023). The preparation is then incubated to permit membrane recovery, followed by purification to remove unloaded cargo. Sonication can increase loading efficiency for both small molecules and some macromolecules, but excessive mechanical stress may alter vesicle size, membrane integrity, or biological activity(Colja et al., 2023).

Another commonly used method is freeze–thaw cycling, in which exosomes and cargo is repeatedly frozen and thawed to induce transient membrane disruption and facilitate cargo entry(Guarro et al., 2025). After several cycles, the preparation is incubated and purified to remove unencapsulated molecules. This method is simple and does not require specialized instrumentation, but repeated freezing and thawing may promote vesicle fusion, aggregation, or structural damage, thereby compromising reproducibility(Ramesh et al., 2025).

In some studies, extrusion has also been used to improve loading efficiency. In this approach, exosomes are mixed with the cargo and repeatedly forced through polycarbonate membranes with defined pore sizes, which can promote membrane rearrangement and cargo incorporation. Although extrusion may improve encapsulation, it can substantially alter vesicle morphology and is therefore best considered when high loading efficiency is prioritized over strict preservation of native vesicle architecture(Guarro et al., 2025).

#### 4.1.2 Chemical loading methods

Chemical loading methods facilitate cargo incorporation by modulating membrane permeability or promoting electrostatic/ionic interactions between the cargo and the vesicle.

A representative example is CaCl₂-mediated loading, which has been used particularly for nucleic acids. In a typical protocol, purified exosomes are mixed with the desired RNA or DNA cargo in the presence of calcium chloride, followed by incubation and, in some cases, a brief heat-shock step to enhance membrane permeability and cargo association. The preparation is then cooled and purified to eliminate free cargo and residual reagents. This approach is relatively simple and may improve loading efficiency compared with passive incubation, but residual salts or incomplete purification can affect downstream biological evaluation(Zhang et al., 2017).

Other chemical approaches employ membrane permeabilizers or related chemical facilitators to promote cargo entry into isolated exosomes. In general, exosomes are incubated with the cargo in the presence of a chemical facilitator, followed by removal of excess reagent and non-encapsulated cargo through purification(Klyachko et al., 2024). These methods can enhance loading of hydrophilic compounds or nucleic acids, but they also raise concerns regarding reagent toxicity, membrane perturbation, and interference with subsequent functional assays(McCann et al., 2022).

In addition, some studies have explored pH- or ion-gradient-assisted loading to facilitate incorporation of specific cargo, particularly weakly basic small molecules(Jeyaram et al., 2020). In such cases, exosomes are exposed to controlled chemical conditions that favor cargo diffusion or retention across the membrane, after which the vesicles are re-equilibrated and purified. While potentially useful for selected drug classes, these methods remain less standardized than physical loading approaches(Kronstadt et al., 2022).

#### 4.1.3 Biological (endogenous) loading methods

Biological loading strategies introduce cargo during exosome biogenesis by engineering or treating donor cells before vesicle collection. Compared with exogenous approaches, biological loading generally better preserves membrane integrity and native surface composition, although it depends strongly on donor-cell biology and intracellular sorting efficiency.

One common endogenous strategy is donor-cell transfection. In a typical workflow, donor cells are transfected with plasmids, miRNA mimics, siRNAs, mRNAs, or protein-expression constructs, and are subsequently cultured under defined conditions to produce cargo-enriched exosomes(Nebogatova et al., 2023). Conditioned medium is then collected, and exosomes are isolated and characterized to confirm cargo incorporation. This method is especially suitable for nucleic acids and engineered proteins, and it often yields vesicles with good structural integrity. However, loading efficiency depends on cellular uptake, expression level, intracellular trafficking, and endogenous sorting mechanisms, which may vary substantially across cell types(Zickler et al., 2024).

A related strategy involves genetic engineering of donor cells to enhance selective cargo packaging. For example, donor cells may be engineered to express fusion proteins, RNA-binding proteins, or sorting motifs that favor recruitment of therapeutic cargo into exosomes during multivesicular body formation(Obuchi et al., 2025). After stable or transient expression is established, exosomes are harvested and analyzed for cargo enrichment. These approaches may offer higher specificity and better control over biological loading, but they increase manufacturing complexity and may raise additional safety and regulatory considerations(Martin Perez et al., 2024; Zickler et al., 2024).

For small-molecule drugs, donor cells can also be preconditioned or incubated with the therapeutic compound, allowing intracellular uptake and subsequent packaging into secreted exosomes. Exosomes are then isolated from conditioned medium and assessed for drug loading. This method can preserve vesicle structure and exploit natural sorting pathways, but drug toxicity to donor cells and variable packaging efficiency remain important limitations(Lan et al., 2023).

#### 4.1.4 Method selection and formulation considerations

No single loading strategy is universally optimal. Method selection should depend on cargo properties, administration route, and translational requirements. Passive incubation and some chemical approaches are generally more suitable for hydrophobic small molecules, whereas electroporation and donor-cell engineering are more often used for nucleic acid cargo. Although sonication, freeze–thaw cycling, and extrusion may improve loading efficiency, they may also compromise vesicle integrity and reproducibility. Therefore, loading strategies should be evaluated not only by loading efficiency, but also by vesicle recovery, physicochemical stability, cargo retention, biological activity, and batch consistency. Representative physical, chemical, and biological cargo-loading methods are summarized in Table 3.

**Table 3:** Representative cargo-loading methods for exosome-based drug delivery systems.

Category	Method	Procedure	Cargo	Advantages	Limitations	Ref.
Physical	Co-incubation	Cargo incubation; free-cargo removal	Hydrophobic small molecules	Operational simplicity; mild processing	Limited loading efficiency	(Nemidkanam et al., 2024)
	Electroporation	Electrical pulsing; post-loading purification	siRNA; miRNA; mRNA	Efficient nucleic acid loading	Aggregation; membrane destabilization	(Torabi et al., 2024)
	Sonication	Sonication-assisted loading; purification	Small molecules; proteins	Enhanced loading efficiency	Mechanical stress; structural alteration	(Colja et al., 2023)
	Freeze–thaw cycling	Repeated freeze–thaw cycling; purification	Small molecules; proteins	Procedural simplicity	Vesicle fusion; low loading efficiency	(Ramesh et al., 2025)
	Extrusion	Membrane extrusion-assisted loading	Drugs; proteins; RNAs	Improved encapsulation	Structural alteration	(Guarro et al., 2025)
Chemical	CaCl₂-mediated loading	CaCl₂-assisted incubation; purification	miRNA; siRNA	Simple operation; enhanced RNA loading	Residual salt interference	(Zhang et al., 2017)
	Chemical facilitator−/cationization-assisted loading	Facilitator-assisted incubation	Hydrophilic drugs; RNAs	Improved loading of difficult cargo	Cytotoxicity; analytical interference	(Klyachko et al., 2024)
	pH−/ion-gradient loading	Gradient-driven loading	Ionizable small molecules	Applicability to selected cargo	Limited standardization	(Kronstadt et al., 2022)
Biological (endogenous)	Donor-cell transfection	Pre-isolation donor-cell transfection	RNAs; proteins	Preserved native vesicle features	Sorting-dependent efficiency	(Zickler et al., 2024)
	Donor-cell engineering	Selective cargo-packaging engineering	RNAs; proteins	Improved loading specificity	Manufacturing complexity	(Martin Perez et al., 2024)
	Drug preconditioning	Pre-harvest drug exposure	Small-molecule drugs	Endogenous sorting utilization	Inconsistent loading	(Lan et al., 2023)

### 4.2 Targeting modification strategies

Beyond improving loading efficiency, targeting modifications enhance the precision of exosome-mediated drug delivery. Although exosomes may achieve some passive accumulation due to stability and prolonged circulation, intrinsic targeting specificity is often insufficient(Potestà et al., 2020; Zhou et al., 2018). This limitation can reduce clinical efficiency and restrict broader therapeutic applications. Consequently, targeted engineering strategies have been developed to increase binding affinity to recipient cells through genetic or chemical modification.

In ADEs, transmembrane proteins such as LAMP and GPI, together with tetraspanins (e.g., CD63, CD9, and CD81), contribute to exosomal membrane architecture and provide anchoring sites for engineering. These proteins can be genetically modified to display ligands that enhance receptor engagement on target cells, thereby improving delivery efficiency(Lopez Baltazar et al., 2024). A widely used strategy fuses targeting peptides to LAMP-2B to facilitate transport across biological barriers, including the blood–brain barrier, improving delivery of drugs or siRNAs(Mentkowski et al., 2018). Representative examples include iRGD-LAMP-2B–modified exosomes for tumor targeting(Tian et al., 2014) and cardiac-targeting peptide–LAMP2B constructs to enhance cardiac tropism(Kim et al., 2018), as well as rabies virus glycoprotein display to improve neuronal delivery(Alvarez-Erviti et al., 2011). Li et al. developed CD9-HuR–functionalized exosomes by transfecting HEK293T cells with a CD9-HuR plasmid, which increased drug loading(Li et al., 2019). Matsumoto et al. also engineered CpG-SAV-Exo using genetic methods for cancer immunotherapy(Morishita et al., 2016).

Chemical conjugation has also been used to anchor targeting moieties onto exosome membranes. For example, click chemistry enables rapid covalent attachment of small or large molecules via copper-catalyzed azide–alkyne cycloaddition and can preserve exosome size and bioactivity(Smyth et al., 2014). Click-based functionalization has been applied to generate peptide-conjugated exosomes for tissue repair(Zeng et al., 2023b). Nevertheless, potential risks such as protein denaturation or exosome aggregation should be evaluated systematically, particularly for translational applications.

Beyond covalent strategies, multiple non-covalent approaches—including multivalent electrostatic interactions, ligand–receptor interactions, hydrophobic interactions, aptamer-based functionalization, and CP05 peptide anchoring—have been explored to generate targeted exosomes(Armstrong et al., 2017; Kooijmans et al., 2016; Lee et al., 2016; Liang et al., 2022; Qi et al., 2016; Wan et al., 2017). Despite these advances, the safety of modified exosomes remains insufficiently characterized; in particular, surface modification may elevate the risk of undesired immune responses. Overall, integrating cargo loading with targeting modification can substantially enhance therapeutic potential by increasing payload capacity and improving site-specific delivery, thereby boosting efficacy and reducing off-target exposure.

### 4.3 Industrialization and regulatory considerations

Beyond laboratory-scale engineering, translation of exosome-based formulations requires alignment with industrial manufacturing and regulatory frameworks. Key challenges include scalable production, quality control, and safety assurance(Wang et al., 2025a).

From a manufacturing standpoint, ADEs rely largely on cell culture–based production, which can limit yield and increase cost. In contrast, PELNs derived from edible or medicinal plants offer potential advantages in scalability and sustainability. As discussed in Section 2.3, both platforms require standardized isolation and purification workflows compatible with good manufacturing practice (GMP), since recovery efficiency, purity, and scalability directly affect downstream quality control and translational feasibility.

Regulatory considerations remain an evolving landscape. Exosome-based products occupy an interface between biologics and nanomedicines, raising questions regarding classification, potency assays, and release criteria. As discussed in Section 2.5, robust characterization is essential for defining the critical quality attributes required for quality control, translational development, and regulatory evaluation. Critical attributes likely to be required for regulatory approval include defined composition, reproducible cargo loading, validated dose metrics, stability profiles, and comprehensive safety evaluation(Bibi et al., 2024). Early integration of these considerations into engineering design is therefore essential to facilitate clinical translation.

### 4.4 Challenges and limitations of exosome-based nanocarriers

Despite their substantial promise as biologically derived delivery systems, exosome-based nanocarriers still face major barriers to reproducible formulation development and clinical translation. A central issue is intrinsic heterogeneity. Vesicle size, cargo composition, membrane markers, and biological activity can vary significantly depending on donor cell type, plant source, culture conditions, and isolation workflow(Naskou et al., 2024). Such variability complicates comparability across studies and makes dose standardization, quality control, and batch consistency difficult to establish.

A second limitation lies in the balance between yield, purity, and scalability. For ADEs, large-scale production remains constrained by the dependence on cell culture, relatively low vesicle yield, and high manufacturing cost(Dong et al., 2020). For PELNs, although scalability is more favorable, complex plant-derived matrices introduce additional purification challenges and increase the risk of co-isolating non-vesicular components, metabolites, and other impurities(Huang et al., 2021). Thus, no currently available isolation strategy fully resolves the trade-off between recovery efficiency, purity, and process scalability.

Another important challenge concerns dose definition and pharmacokinetic interpretation. Exosome-based formulations are still reported using inconsistent dose metrics, including particle number, protein amount, and cargo quantity, which limits cross-study comparison and complicates dose definition, exposure–response analysis, and translational interpretation. In addition, biodistribution, intracellular trafficking, and cargo release remain insufficiently quantified in many studies, thereby limiting the establishment of robust PK–PD relationships for therapeutic development(Driedonks et al., 2022; Rosenkrans et al., 2024).

Engineering strategies also introduce important limitations. Although cargo loading and surface modification may improve payload control and targeting precision, they can also compromise vesicle integrity, alter membrane properties, increase aggregation risk, or introduce additional safety concerns(Guarro et al., 2025; McCann et al., 2022). In particular, high loading efficiency may be achieved at the expense of structural stability, while targeting modification may increase immunogenicity or reduce reproducibility during manufacturing.

Post-preparation stability is another major barrier. Exosome formulations are sensitive to storage conditions, freeze–thaw cycles, and processing stresses, all of which may affect vesicle integrity, cargo retention, and biological activity(Trenkenschuh et al., 2022). These issues are especially important for translational development, where long-term storage, transport, and lot release require robust and reproducible stability profiles(Bui et al., 2022; Ren et al., 2025).

These challenges do not diminish the therapeutic potential of exosome-based systems, but instead delineate the principal priorities for future development. Safety and regulatory uncertainty remain important unresolved barriers. Potential risks include off-target biodistribution, unintended immune activation, transmission of undesirable bioactive cargo, and incompletely defined impurity profiles(Gu et al., 2022; Rosenkrans et al., 2024). For PELNs, a further challenge is to distinguish vesicle-mediated pharmacological activity from effects attributable to co-isolated plant-derived metabolites. Taken together, these limitations suggest that the continued development of exosome-based nanocarriers will require not only improved engineering strategies, but also stronger emphasis on standardization, critical quality attributes, quantitative pharmacology, long-term stability control, and modification-specific safety evaluation.

## 5 Clinical trials

Exosome-based therapies have entered early-phase clinical trials, highlighting growing translational momentum. Current studies are investigating the safety, feasibility, and efficacy of natural and engineered extracellular vesicles derived from mammalian and plant sources. Representative trials are summarized in Table 4.

**Table 4:** Ongoing Clinical Trials with Exosomes.

NCT Number	Condition	Phase	type of EV	Modification	Status
NCT04388982	Alzheimer's disease	I/II	Adipose MSC-Exos	None	Recruiting
NCT05152394	Acute kidney injury	I	UC-MSC-Exos	None	Recruiting
NCT06755021	Hepatocellular carcinoma	Not listed	UC-MSC-Exos	None	Recruiting
NCT05158101	Ischemic stroke	I	UC-MSC-Exos	None	Recruiting
NCT01668849	Oral mucositis (cancer)	I	Grape-derived PELNs	None	Completed
NCT01294072	Colorectal cancer	I	Ginger-derived PELNs	Curcumin loading	Recruiting
NCT04879810	Inflammatory bowel disease	I	Ginger-derived PELNs	+/ - Curcumin	Completed
NCT03384433	Acute ischemic stroke	I/II	BM-MSC-Exos + miR-124	miR-124 enrichment	Completed

Most trials focus on mesenchymal stem cell–derived exosomes (MSC-Exos) owing to their established immunomodulatory and regenerative properties. For example, NCT04388982 evaluates intranasal delivery of adipose-derived MSC-Exos for Alzheimer's disease. NCT05152394 and NCT06755021 assess intravenous administration of umbilical cord–derived MSC-Exos (UC-MSC-Exos) in acute kidney injury and hepatocellular carcinoma, respectively. In addition, NCT05158101 investigates intranasal UC-MSC-Exos for ischemic stroke.

PELNs have also advanced into clinical trials and are primarily administered orally. For example, GDNs were evaluated for prevention of radiotherapy-induced oral mucositis (NCT01668849). GELNs, with or without curcumin loading, are being investigated for colorectal cancer (NCT01294072) and inflammatory bowel disease (NCT04879810).

Engineered exosomes represent an emerging frontier in exosome therapeutics. Notably, NCT03384433 used bone marrow–derived MSC-Exos enriched with miR-124 via stereotactic injection for acute ischemic stroke, demonstrating acceptable safety and preliminary functional signals. This study highlights the potential of RNA-engineered exosomes for targeted neural repair.

## 6 Conclusion and perspectives

Exosomes and exosome-like nanoparticles constitute a promising class of biologically sourced nanomedicines whose lipid bilayer architecture enables protection of labile biomacromolecules and supports interaction with complex biological barriers. A key message emerging from current evidence is that therapeutic effects are frequently cargo-driven, yet successful translation requires treating exosomes as pharmaceutical products—where performance depends on controllable formulation attributes rather than biological plausibility alone. Accordingly, future progress should prioritize development frameworks that quantitatively integrate administered dose, in vivo exposure (biodistribution/PK), cellular delivery efficiency, and pharmacodynamic response, while accounting for the intrinsic heterogeneity of vesicle populations.

From a product-development perspective, the field would benefit from (i) harmonized dose units and normalization strategies (e.g., particle number, total protein, and cargo copy number) and explicit reporting standards to enable inter-study comparability; (ii) robust CQAs and potency assays that reflect intended mechanisms (e.g., cargo integrity, membrane composition, functional uptake, and downstream pathway modulation) and support lot release; (iii) systematic evaluation of stability and trafficking (including corona formation, opsonization, and endosomal escape) as determinants of effective intracellular bioavailability; and (iv) manufacturing solutions compatible with GMP scale-up, including reproducible isolation, purification, sterile processing, and long-term storage without loss of function.

PELNs are particularly attractive for pharmaceutics because they combine renewable sourcing, scalability, and oral delivery potential, but their translation will hinge on addressing compositional variability arising from plant species, growth conditions, and isolation workflows, as well as disentangling carrier effects from co-encapsulated metabolites. For engineered systems, improved targeting and loading must be balanced against risks of altered immunogenicity, aggregation, or compromised stability, underscoring the need for modification-specific safety packages and comparability protocols.

Overall, positioning exosome-based platforms—especially PELNs for gastrointestinal delivery—as clinically deployable formulations will require a shift toward quantitative, CMC-driven development. Integrating multi-omics compositional profiling with exposure–response modeling and standardized release criteria should enable more predictable design, facilitate regulatory acceptance, and accelerate the transition from preclinical promise to reproducible therapeutic products.

## Funding

This research was funded by the 10.13039/501100001809National Natural Science Foundation of China (32002244), and the Major Project of Zhejiang Provincial Natural Science Foundation (LHDMD26E030001).

## Ethics approval and consent to participate

Not applicable.

## Consent for publication

Not applicable.

## CRediT authorship contribution statement

**Hongfeng Xu:** Writing – original draft, Visualization, Investigation, Data curation. **Jin Zhang:** Writing – review & editing, Supervision, Funding acquisition. **Meng Li:** Writing – review & editing, Supervision, Funding acquisition.

## Declaration of competing interest

The authors declare that they have no competing interests.

## Data Availability

No data was used for the research described in the article.
